# Selenium Improved Phenylacetic Acid Content in Oilseed Rape and Thus Enhanced the Prevention of *Sclerotinia sclerotiorum* by Dimethachlon

**DOI:** 10.3390/jof8111193

**Published:** 2022-11-11

**Authors:** Huan Zhang, Qin Cheng, Xu Wang, Wei Jia, Jiatao Xie, Guocheng Fan, Chuang Han, Xiaohu Zhao

**Affiliations:** 1 State Key Laboratory of Agricultural Microbiology, College of Resources and Environment, Huazhong Agricultural University, Wuhan 430070, China; 2 Institute of Quality Standard and Monitoring Technology for Agro-Products of Guangdong Academy of Agricultural Sciences, Guangzhou 510640, China; 3 Fujian Key Laboratory for Monitoring and Integrated Management of Crop Pests, Fuzhou 350003, China

**Keywords:** *Sclerotinia sclerotiorum*, oilseed rape, inhibition, dimethachlon, phenylacetic acid, gene expression

## Abstract

*Sclerotinia sclerotiorum* is a broad-spectrum necrotrophic phytopathogen that can infect many plant species worldwide. The application of fungicides is a common measure for controlling *Sclerotinia sclerotiorum*. Due to the risk of developing resistance to fungicides, it is imperative to find ways to be environmentally friendly and even effective. Using bioactive compounds in plants to reduce the amounts of fungicides has become a clean and sustainable strategy of controlling *Sclerotinia sclerotiorum*. Our study found that selenium in soil mediated the phenylacetic acid-related metabolic pathway in oilseed rape and reduced the incidence rate of *Sclerotinia sclerotiorum*. The growth-inhibition rates of *Sclerotinia sclerotiorum* were observed at 25.82%, 19.67%, and 52.61% for treatments of 0.8 mg·L^−1^ dimethachlon, 0.1 mg·mL^−1^ phenylacetic acid, and dimethachlon (0.8 mg·L^−1^) + phenylacetic acid (0.1 mg·mL^−1^), respectively. Phenylacetic acid reduced the application amount of dimethachlon and enhanced the inhibition effect for *Sclerotinia sclerotiorum*. Results also suggested that phenylacetic acid severely damaged the morphological structure, changed the electrical conductivity, and reduced the capacity of acid production and oxalic acid secretion of *Sclerotinia sclerotiorum* mycelium. Further studies revealed that phenylacetic acid increased the gene-expression level of *Ssodc1*, *Ssodc2*, *CWDE2* and *CWDE10* in mycelium while decreasing the expression level of *SsGgt1*, and phenylacetic acid + dimethachlon reduced the relative expression level of *SsBil*. These findings verified that phenylacetic acid could partially replace the amount of dimethachlon, as well as enhance the prevention of *Sclerotinia sclerotiorum* by dimethachlon, which provides evidence for developing an environment-friendly method for *Sclerotinia sclerotiorum* control.

## 1. Introduction

*Sclerotinia sclerotiorum* (*S. sclerotiorum*) is an aggressive pathogenic fungus with a broad host range and a worldwide distribution that can infect over 400 plant species, including many economically important crops and vegetables, such as rapeseed, sunflower, common bean, soybean, and canola [1,2]. Sclerotinia stem rot (SSR), caused by Sclerotinia, is the primary fungal disease of rapeseed, which severely affects the yield and quality of rapeseed and endangers the production safety of rapeseed in many areas of Canada, America and China [3].

Recently, the methods for controlling plant diseases caused by *S. sclerotiorum* have mainly focused on agronomic regulation and bio-chemical control [4,5]. *S. sclerotiorum* has a wide host range and no host specificity, therefore, many agricultural practices, such as crop rotation, timely sowing, drainage of drainage ditches and rational fertilization are effective methods against *S. sclerotiorum* [6]. However, agricultural control cannot permanently control sclerotinia disease because sclerotia is resistant to stress and crop rotation. In addition, biological control is a common strategy but is unstable in field-crop sclerotinia-disease prevention and control. Currently, chemical agents are used primarily for the prevention and control of sclerotinia [7]. Unfortunately, with the frequent use of fungicides, sclerotia developed resistance to some commonly used chemical fungicides, leading to a decline in the effect of chemical control, for example, sclerotia strains resistant to dimethachlon (DIM) [8]. Furthermore, environmental pollution and pesticide residues caused by unscientific application of chemical pesticides are becoming increasingly serious. Therefore, reducing the use of pesticides and using plant-derived substances, which is a clean and sustainable strategy, as well as low-cost plant disease control, have increasingly attracted attention.

Recently, compound preparation has played an important role in improving the effectiveness of *S. sclerotiorum* control, expanding the germicidal spectrum, reducing the dosage, delaying the drug resistance of pathogenic bacteria, and prolonging the service life and health of fungicides [9]. The use of fungicides for biologically active compounds of microbial or plant origin together could become a promising approach in crop protection [10,11,12]. The resulting synergistic or additive effect of both components provided a significant reduction in the efficient concentrations of chemical fungicides. For instance, a high fungicidal activity of the mycelial of *P. chrysogenum* F-24-28 (DMP) was demonstrated [13]. In addition, a combined application of DMP (0.3 g·L^−1^) and azoxystrobin at low dosage (2.5 mg·L^−1^) showed a high suppressing activity towards *S. sclerotiorum* (even 100% growth inhibition), including inhibition of a sclerotia formation [14]. 

Many studies have shown that Se can alleviate the damage to plants caused by stress conditions such as heavy metal toxicity, salt damage, drought, and diseases [15,16]. The application of Se in plant protection can improve the yield and quality of crops while ensuring agricultural safety [17]. Under in vitro culture conditions, Se has been shown to inhibit the growth of a variety of plant pathogens [18,19]. A previous study conducted by our group indicated that up-regulation of metabolite (PA) content derived from rape straw pretreated with selenium (Se) in soil improved the inhibition of *S. sclerotiorum* growth [20]. Another study found that PA could be a good alternative chemical reagent to replace the use of DIM [21]. However, the key metabolic pathways affected by Se-mediated up-regulation of PA in dissolved organic matter (DOM) from rape straw were not clear, and whether these metabolic pathways were directly related to the incidence rate of *S. sclerotiorum* needs to be further revealed. Moreover, PA, as a key metabolite in various metabolic pathways of rape, is also a compound of plant origin. How much DIM can be replaced by PA, as well as the action mechanism, needs to be revealed further. 

Therefore, the objectives of this study were: (1) to clarify the effect of the PA-related metabolic pathway mediated by Se on the reduction incidence rate of *S. sclerotiorum*; (2) to examine the effect of PA enhancing the prevention of *S. sclerotiorum* by DIM; (3) to illuminate the structure change and biochemical responses of mycelia to PA and DIM, and (4) to investigate the change of pathogenicity gene-expression level in mycelia when treated with PA and DIM. All of these studies could help us to reveal the effect of PA on the growth of *S. sclerotiorum*. The results could provide a novel method and evidence for reducing the use of fungicides and improving the effects of *S. sclerotiorum* control.

## 2. Materials and Methods

### 2.1. Pathogens and Reagent Preparation

*S. sclerotiorum* (JZJL-13) was obtained from the Key Laboratory of Crop Disease Monitoring and Safety Control, College of Plant Science and Technology, Huazhong Agricultural University. To obtain a new mycelium of S. sclerotiorum, the blade was sterilized and used to cut the sclerotia, then the sclerotia was placed into the medium. After growing the sclerotia, taking the distance of mycelium at the same radius as the medium, we then inoculated it onto a new PDA and incubated at 23 °C for 48 h. 

PA was an analytical reagent purchased from Guangzhou Card Finn Biological Technology Co., Ltd. (Guangzhou, China). DIM, as wettable powder with an effective content of 40%, was purchased from Zhejiang Shengtong Bio Chemical Limited Co., Ltd. (Zhejiang, China). PA and DIM were selected in this study based on the results.

### 2.2. Metabolite Note and KEGG Pathway Analysis 

A field trial with Se treatments (0, 1.12 kg·ha^−1^) was conducted, rape straw was collected at the mature stage, DOM from rape straw (RSDOM) was obtained, and the RSDOM metabolites of Se_0_ and Se_1.12_ treatment were investigated by gas chromatography-time-of-flight mass spectrometry [20]. On this basis, to illustrate the relationship between the metabolic pathway of rape regulated by Se and disease resistance further, the metabolites were noted and the KEGG pathway of RSDOM was analyzed with and without Se treatment.

### 2.3. EC50 of DIM and PA

The effective medium concentrations (EC_50_) of DIM and PA were determined by the mycelial growth rate method first. EC_50_ represents a concentration of 50% of maximal effect. DIM and PA solutions were added to PDA medium, the final series concentrations of DIM in PDA were 0, 0.5, 1.0, 2 mg·L^−1^, and the final series concentrations of PA were 0, 0.05, 0.1, 0.2, 0.4 mg·mL^−1^. After the medium was cooled and solidified, the mycelium obtained from pathogen preparation was inoculated in the center of the medium. After that, the PDA plate was put upside down in a 23 °C incubator for 48 h. Then, the diameter of each colony was measured, and the inhibition rate and EC_50_ value were calculated.

### 2.4. Mycelial Growth Assay and Sclerotial Formation

To examine the effect of PA and DIM on the growth of *S. sclerotiorum,* 0.8 mg·L^−1^ DIM and 0.1 mg·mL^−1^ PA were chosen according to the results of EC_50_ and previous study [16]. The effects of DIM and PA on mycelial growth of *S. sclerotiorum* were assayed in PDA medium with different treatments (CK, 0.8 mg·L^−1^ DIM, 0.1 mg·mL^−1^ PA, 0.8 mg·L^−1^ DIM + 0.1 mg·mL^−1^ PA). Growth of *S. sclerotiorum* was measured after incubation at 23 °C in darkness for 36 h. The inhibitory ratio (%) = (d_CK_ − d_treated_)/d_CK_. “d_CK_” was the treatment without DIM and PA. “d_treated_” was the treatment with DIM or PA. Each treatment was set for three repetitions, and two identical groups were set to analyze sclerotial formation. To record the number of sclerotia on each PDA plate, each plate was incubated at 23 °C in the dark for 10 d. Then, all the sclerotia on each plate were moved into empty plates, and the fresh weight was recorded.

### 2.5. Measurement of Pathogenicity on Detached Leaves of Rape 

The pathogenicity of *S. sclerotiorum* was studied on detached leaves of oilseed rape. The leaves were picked from the field experiment that was performed at the eco-agriculture base (30°28′26″ N, 114°2′15″ E), Huazhong Agricultural University, Wuhan, China. Leaves were inoculated with PDA mycelial plugs that were produced by a different treatment (the treatments were the same as the above). In order to help facilitate the infection of mycelia into leaves, all leaves were the same size and wounded with sterile needle tips before inoculation. Then, the leaves were incubated in a growth chamber (23 °C, relative humidity 80%). After 36 h, lesion diameters on each leaf were recorded.

### 2.6. Scanning Electron Microscopy (SEM) and Transmission Electron Microscopy (TEM) Analysis 

The membrane structure changes of mycelia response to PA and DIM were determined by SEM and TEM. *S. sclerotiorum* mycelium was cultured with and without treatment on PDA medium that was covered with cellophane in an incubator of 23 °C for 48 h. Then, glass paper was cut into small pieces and placed in tubes containing glutaraldehyde solution. After 24 h, gradient alcohol series (30%, 50%, 70%, 80%, 90%, 100%) were used only for dehydration [22]. Next, mycelium samples were dried with a critical point dryer in Wuhan (Hitachi HCP-2 from Japan) for 0.5 h. The dried samples were sputter-coated with gold for 100 s at 20 mA using a low-vacuum coater in Wuhan (Leica EM ACE 200 from Germany), generating an approximately 10 nm coating thickness. Finally, mycelia were observed with SEM (Hitachi SU-8010) under an accelerating voltage of 3.0 KV. The TEM (TEM, H-7650, Hitachi, Japan) observation was taken according to a modification method [23]. Sclerotia was collected from four treatments grown at 23 °C for 15 d on PDA medium. Samples were fixed with 2.5% glutaraldehyde in 0.1 M phosphate buffer (pH = 7.2) for 4 h at 4 °C and shook several times, followed by a thorough rinse with phosphate buffer for 4 h. Then, the samples were placed into 1% osmium tetroxide at 4 °C for 2 h. After that, the samples were dehydrated in graded acetone series for 4 h and were allowed to immerse in a mixture solution with grade acetone and resin for 4 d. Samples were cut using a Leica Ultracut UCT ultramicrotome and collected into 200-mesh copper grids. After that, samples were dyed with uranyl-acetate and lead citrate for 30 min, and the grids were examined using TEM under an accelerating voltage of 80 kV.

### 2.7. Electrical Conductivity Assay

The electrical conductivity of the mycelia was measured [24]. Cultivated in PDA medium for 3 days, mycelial plus (5 mm in diameter) from fresh edges of 3-day-old colonies were transferred into 50 mL PDB medium and incubated at 23 °C for 7 days. Mycelia were supposed to be centrifuged at 4000 rpm for 10 min and washed three times with sterilized water. Then, 3 g of mycelia was placed into tubes containing DIM or PA solutions. The electrical conductivity of the solution was monitored by an electrical conductivity meter (DDS-307A, Shanghai Leici Instrument Inc., Shanghai, China) at 0, 10, 20, 30, 60, 120, 180 min, respectively. Three replicates were performed for each treatment.

### 2.8. Acid Production Determination 

The effect of DIM or PA on acid production was determined by PDB medium study, following the method with slight modification [24]. Mycelial plugs (6 mm diameter) were cut from fresh edges of 2-day-old colonies in PDA medium. Then, the samples were transferred into a 50 mL flask with PDB medium with different treatments (CK, DIM, PA, DIM + PA) and incubated at 23 °C in the dark for 48 h. Each 50 mL flask contained five mycelial plugs. Subsequently, the PDB solution was centrifuged (5000× *g*, 5 min) and the acid of the liquid was determined by the Seven2Go pH meter S2-Std-Kit (Mettler Toledo instruments Co., Ltd., Shanghai, China). The pH in PDB medium was measured to investigate the change of acid production in mycelium due to different treatments.

### 2.9. Oxalic Acid (OA) Content Measurement 

OA content measurements were carried out with *S. sclerotiorum* mycelium that was cultured in potato dextrose broth (PDB) medium containing different treatments (CK, DIM, PA, DIM + PA). The supernatant was used to identify the OA content [20]. The OA content was identified using a colorimetric method. Briefly, 0.4 mL supernatant was moved to a tube with 0.1 mL 0.5 mg· mL^−1^ Fe^3+^ standard solutions, 1 mL KCl-HCl solution (3.7 g·L^−1^ KCl and 5.4 g·L^−1^ HCl, pH 2.0) and 0.06 mL 0.5% sulfosalicylic acid (*w*/*v*). After 20 min, the absorbance at 510 nm was read from a UV-5200 ultraviolet spectrophotometer, and sodium oxalate served as the standard. Each treatment contained three replicates.

### 2.10. RNA Isolation and Quantitative Real-Time PCR (qRT-PCR) Analysis

The relative gene-expression levels of six pathogenic genes of *S. sclerotiorum* (*Ssodc1, Ssodc2, CWDE2, CWDE10, SsBil1, SsGgt1*) were assessed by qRT-PCR. Target primer sequences are given in Table 1. *S. sclerotiorum* mycelium was obtained with different treatments (CK, 0.8 mg·L^−1^ DIM, 0.1 mg·mL^−1^ PA, 0.8 mg·L^−1^ DIM + 0.1 mg·mL^−1^ PA), and each treatment contained five replicates. Total RNA was extracted using NI-*Sclerotinia sclerotiorum* RNA Reagent (Newbio Industry, Tianjin, China), then RNA was reverse transcribed with reagent from Trans Gen Biotech, Beijing (EasyScript One-Step gDNA Removal and cDNA Synthesis SuperMix). qRT-PCR was performed using an ABI Q6 Flex system (Applied Biosystems, USA). Each sample was repeated three times. The relative expression level of target genes was calculated by 2^−ΔΔCT^ method. 

### 2.11. Statistical Analysis

GC-TOF-MS analysis was conducted [25]. Chroma TOF 4.3X Software (Leco), (Carl Schultz, America) equipped with the Leco-Fiehn Rtx5 database was used to identify metabolites. All data analysis was performed with SPSS software version 22.0 and the results were reported as the mean ± standard error (S.E.) of three replicates. One-way analysis of variance (ANOVA) was carried out to analyze the results, which included mycelial growth assay. Differences between different treatments were subjected to the LSD test by Duncan’s multiple comparison (*p* < 0.05). Commercial databases including KEGG http://www.genome.jp/kegg/ (accessed on 1 March 2019) and MetaboAnalyst http://www.metaboanalyst.ca/ (accessed on 1 March 2019) were utilized to search for the pathways of metabolites.

## 3. Results

### 3.1. Metabolic Pathway Analysis

**Figure 1:**
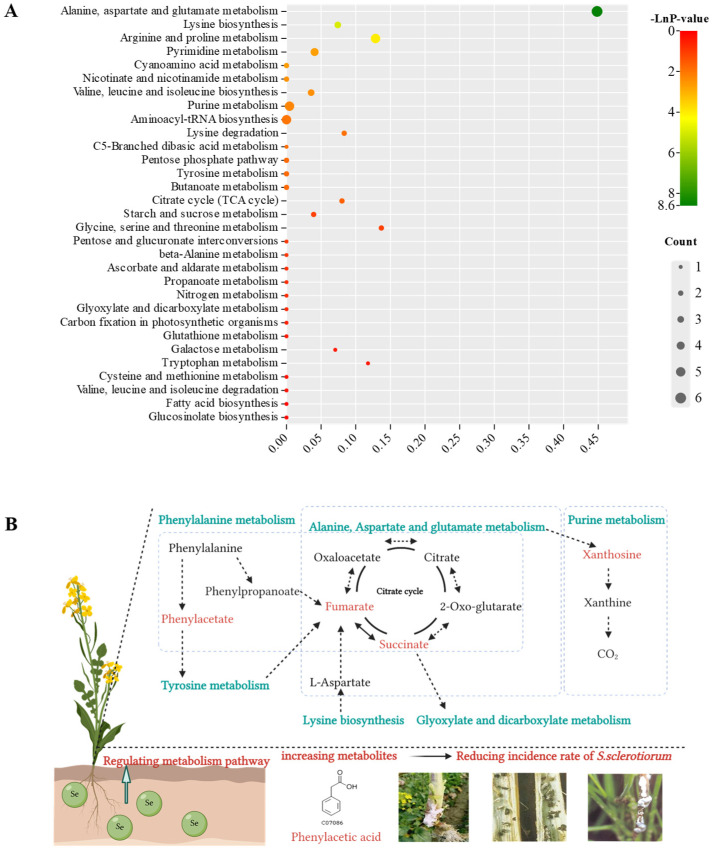
(**A**) KEGG metabolic pathway analysis for DOM from rape straw between group Se_0_ and Se_1.12_. The darker the color, the smaller the P value and the more significant the enrichment degree. (**B**) A schematic diagram showing that Se regulated the metabolic pathway that is relevant to disease resistance and reduced the incidence rate of *S. sclerotiorum*. Se regulated multiple metabolism pathways and specific metabolites, which were relevant to the effects of systemic resistance.

Differential metabolites were shown in our study, PA content derived from rape straw was up-regulated with Se added into soil [20]. KEGG pathway enrichment analysis was conducted according to the consequences of differential metabolites (Table 2). Differential metabolites were enriched in 35 pathways, and Se mainly regulated phenylalanine metabolism, alanine, aspartate and glutamate metabolism, lysine biosynthesis, arginine and proline metabolism and the purine metabolism pathway (Figure 1A). PA was mainly involved in tyrosine metabolism and phenylalanine metabolism pathways. A schematic diagram was structured showing that Se mediated the PA-related metabolic pathway and reduced the incidence rate of *S. sclerotiorum* (Figure 1B).

### 3.2. Inhibitory EC_50_ of PA and DIM on S. sclerotiorum 

**Figure 2:**
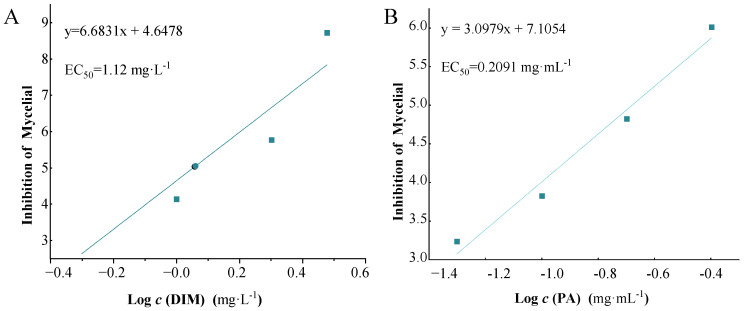
**Inhibitory effects and inhibitory concentration of DIM and PA for *S. sclerotiorum*.** (**A**) Baseline of DIM sensitivity to *S. sclerotiorum.* (**B**) Baseline of PA sensitivity to *S. sclerotiorum*.

The *S. sclerotiorum* inhibitory EC_50_ of DIM and PA were determined. The inhibitory effects of DIM and PA on *S. sclerotiorum* growth are presented in Figure 2A,B. The DIM and PA EC_50_ of *S. sclerotiorum* were 1.12 mg·L^−1^ and 0.2091 mg·mL^−1^, respectively. In the previous study, the inhibitory ratio of *S. sclerotiorum* was 35.5% and 68.73% with 1 mg· L^−1^ DIM and 1 mg·L^−1^ DIM + 0.1 mg·mL^−1^ PA treatment, respectively [16]. 

Combining with the results of EC_50_, the amount of DIM was reduced; 0.8 mg·L^−1^ DIM and 0.1 mg·mL^−1^ PA were chosen to investigate the inhibition of *S. sclerotiorum* growth, morphological structure changes of *S. Sclerotiorum*, biochemical responses of mycelia and pathogenic gene-expression levels further. 

### 3.3. Effects of PA on S. sclerotiorum Growth and Sclerotial Formation 

**Figure 3:**
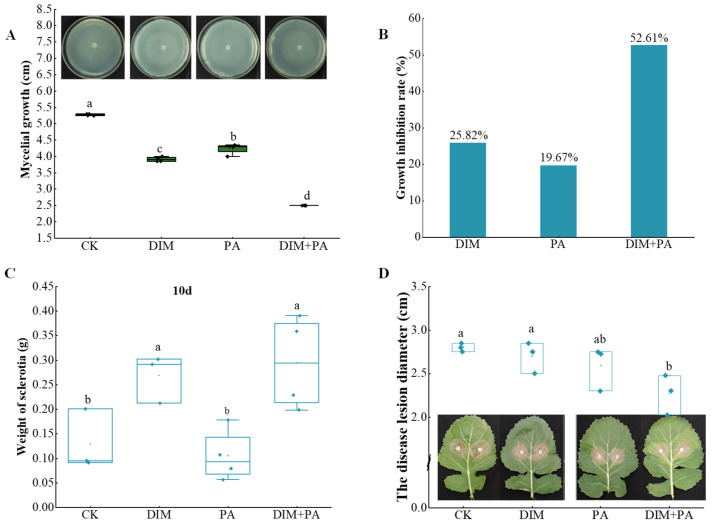
**Inhibition of *S. sclerotiorum* growth by DIM and PA**. (**A**) The disease lesion diameter of rape leaves in vitro under treatment. (**B**) Growth-inhibition rate of *S. sclerotiorum*. (**C**) Effect of DIM and PA on weight of sclerotia after 10 days. (**D**) Effects of DIM and PA on the pathogenicity of rape leaves in vitro. Different letters (a, b, c, d) indicate statistically significant differences among the different treatments (*p* < 0.05). The treatments were: CK, DIM (0.8 mg·L^−1^), PA (0.1 mg·mL^−1^), and DIM (0.8 mg·L^−1^) + PA (0.1 mg·mL^−1^), respectively.

Compared with the control, all the treatments significantly inhibited the growth of *S. sclerotiorum* (Figure 3A), while DIM + PA and DIM showed higher growth-inhibition rates than PA. The growth-inhibition rates were 25.82% and 19.67% with treatment of 0.8 mg·L^−1^ DIM and 0.1 mg·mL^−1^ PA, respectively (Figure 3B). Compared with the treatment of 1 mg·L^−1^ DIM, the growth-inhibition rate increased from 35.5% to 52.61% with 0.8 mg·L^−1^ DIM + 0.1 mg· mL^−1^ PA. In other words, 0.1 g of PA in 1L solution could replace 20% of DIM and increase the 17.11% growth-inhibition rate of *S. sclerotiorum* (Table 3).

In addition, the weight of sclerotia with different treatment after 10 d was determined. Interestingly, compared with the control, the weights of sclerotia in the treatments of DIM and DIM + PA were increased by 37.72% and 73.52%, respectively (Figure 3C). 

### 3.4. Effect of PA on Lesion Development on Detached Leaves

As shown in Figure 3D, the lesion diameter on leaves treated with PA was significantly decreased by 7.4%, while the lesion diameter on leaves showed no significant difference with DIM. Using DIM and PA together inhibited the virulence of mycelia significantly (*p* < 0.05), and the disease lesion diameter was decreased by 18.9% when compared with the control.

### 3.5. Morphological Structure Changes of S. sclerotiorum 

**Figure 4:**
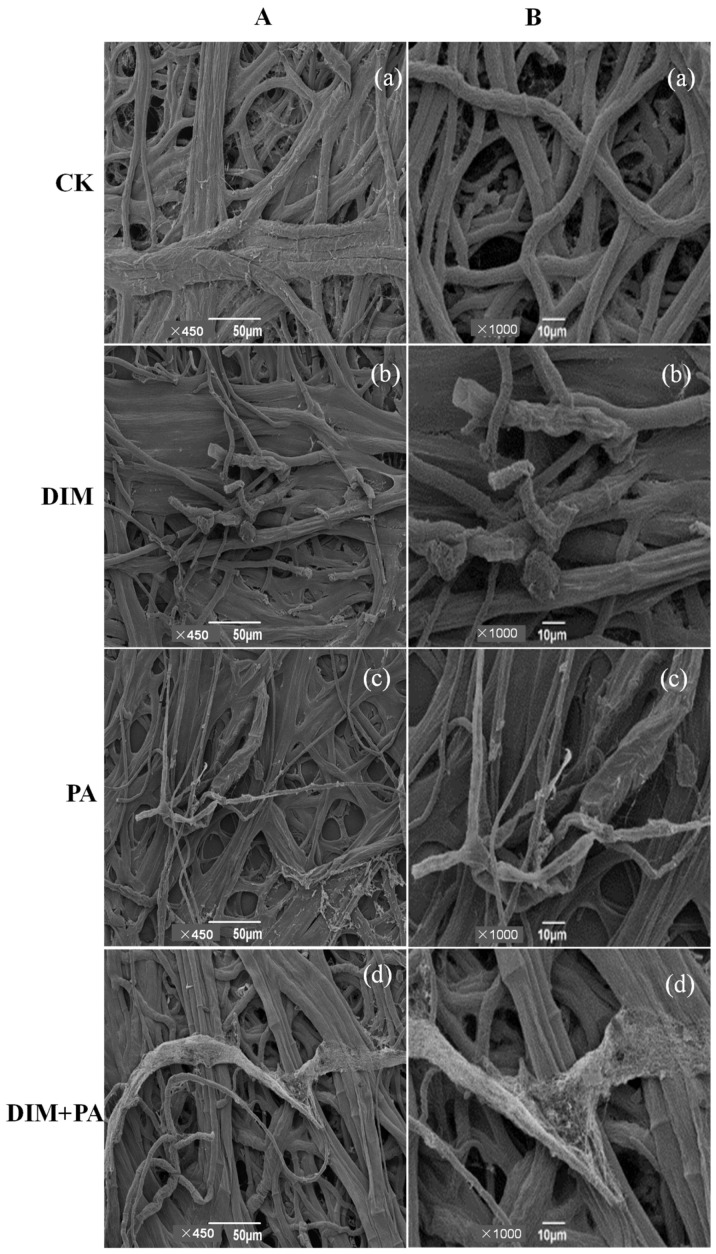
**Effect of DIM or PA treatments on structural appearance changes of sclerotia.** Scanning electron microscope (SEM) images of mycelia in *S. sclerotiorum* receiving DIM or PA. ((**A**): bars = 50 μm, ×450; (**B**): bars = 10 μm, ×1000). The treatments were: (**a**): CK, (**b**): DIM (0.8 mg·L^−1^), (**c**): PA (0.1 mg·mL^−1^), and (**d**): DIM (0.8 mg·L^−1^) + PA (0.1 mg·mL^−1^), respectively.

**Figure 5:**
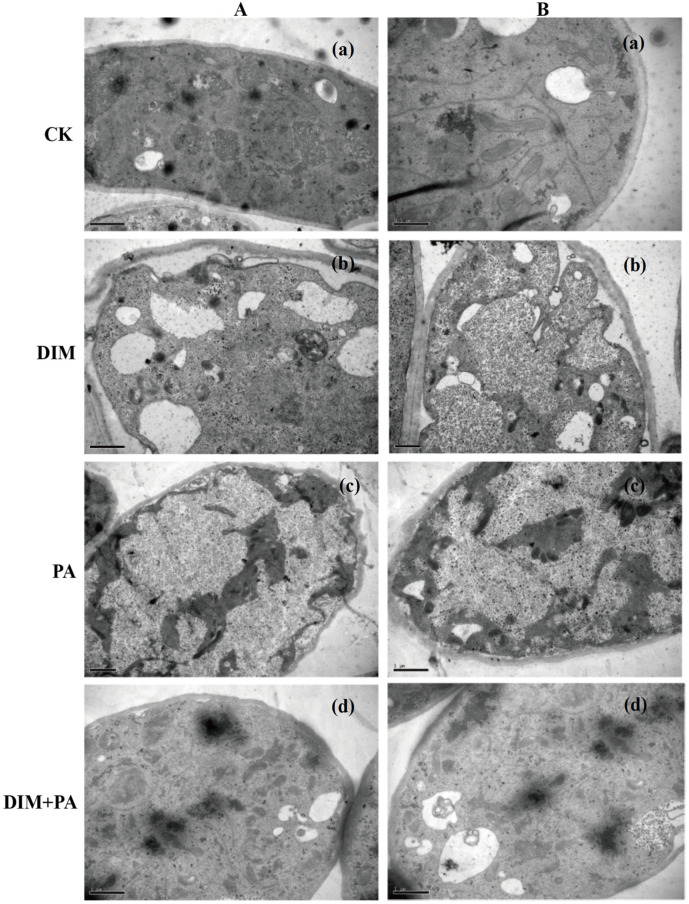
**Effect of DIM or PA treatments on ultrastructural changes of sclerotia.** Representative transmission electron microscopy (TEM) images of sclerotia cells selected from 2 specimens (**A**,**B**) in each treatment. Bar = 1 μm. The treatments were: (**a**): CK, (**b**): DIM (0.8 mg·L^−1^), (**c**): PA (0.1 mg·mL^−1^), and (**d**): DIM (0.8 mg·L^−1^) + PA (0.1 mg·mL^−1^), respectively.

The surface of mycelia in the control treatment was smooth and the connection between mycelia was normal from the scanning electron microscope (Figure 4(Aa,Ba)). After DIM treatment, mycelia were twisted and fractured (Figure 4(Ab,Bb)), while after PA treatment, mycelia were twisted (Figure 4(Ac,Bc)). When treated with DIM and PA, mycelia were shrunk and torn (Figure 4(Ad,Bd)). The mycelia’s inner structure was observed using TEM. The cell wall around the inner cells of untreated mycelia was round and regular in shape, and the outer side of the cell wall was separated from other cells by the intercellular layer (Figure 5(Aa,Ba)). When treated with DIM or PA, the cytoplasm contracted and aggregated, and the cell wall was deformed (Figure 5b,c). After DIM and PA treated together, the cytoplasm was degraded and the cell wall was cracked (Figure 5d). 

### 3.6. Electrical Conductivity, Acid Production and Oxalic Acid Content of Mycelia Analysis

**Figure 6:**
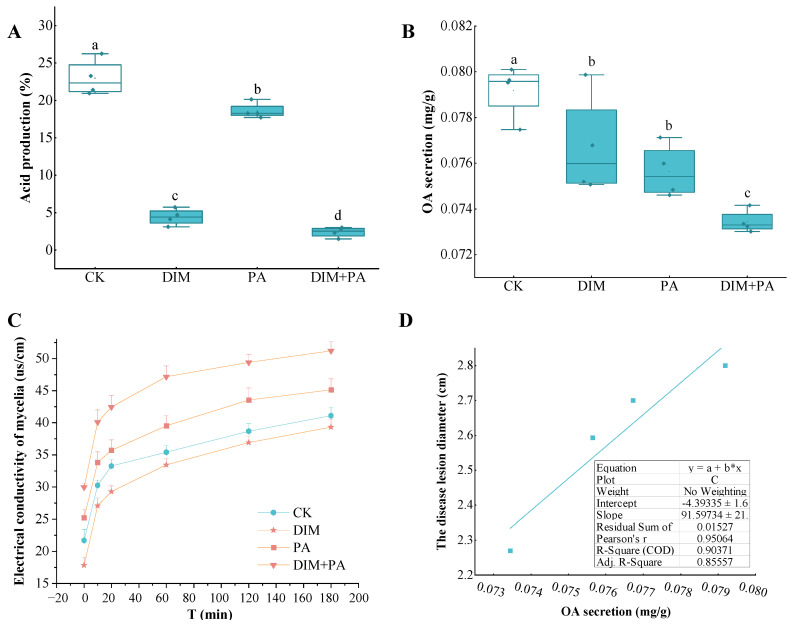
**Effect of Se on the physiological property of S. *sclerotiorum* mycelia.** The treatments were: CK, DIM (0.8 mg·L^−1^), PA (0.1 mg·mL^−1^), and DIM (0.8 mg·L^−1^) + PA (0.1 mg·mL^−1^), respectively. (**A**) The electrical conductivity of *S. sclerotiorum* mycelia. (**B**) Acid production of *S. sclerotiorum* mycelia. (**C**) OA secretion of *S. sclerotiorum* mycelia. (**D**) The correlation between OA secretion and the disease lesion diameter. Vertical bars indicate standard deviations (SD). Different letters (a, b, c, d) indicate statistically significant differences among the different treatments (*p* < 0.05).

Compared with the control, both DIM and PA significantly inhibited the hydrogen production capacity of mycelia, which was decreased by 80.8%, 19% and 89.6%, respectively. The inhibition effect was stronger when DIM and PA were used together (Figure 6A). Compared with the control, both DIM and PA inhibited the oxalic acid secretion of mycelia; similarly, PA obviously enhanced the inhibition effect when it was used together with DIM. The oxalic acid (OA) secretions were decreased by 3.1%, 4.4% and 7.3%, respectively (Figure 6B). As shown in Figure 6C, the electrical conductivity was increased in 120 min and remained constant after 120 min. Additionally, compared with the control, the electrical conductivity of *S. sclerotiorum* mycelium under PA and DIM + PA treatments increased by 12.6% and 27.8% at 120 min, respectively. However, it decreased with DIM treatment. It is speculated that DIM at low concentration thickens the mycelium cell wall by membrane exostosis and making substances accumulate, thus reducing the permeability of the cell membrane. The correlation analysis between the disease lesion diameter and OA secretion showed that the Pearson product-moment correlation coefficient was 0.95064, and the R square was 0.9037 (Figure 6D).

### 3.7. Changes of Pathogenic Gene-Expression Levels 

**Figure 7:**
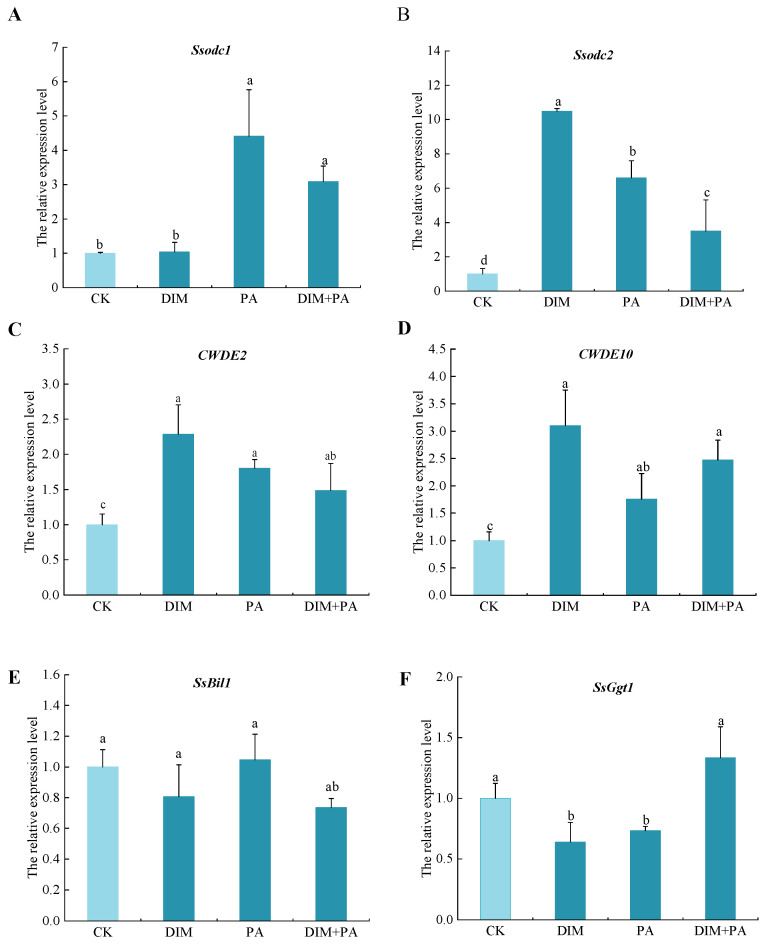
Relative expression level of six target genes of *S. sclerotiorum mycelia* incubated for 48 h in PDA medium containing different treatments. Different bars with different letters (a, b, c) are significantly different (*p* < 0.05). The treatments were: CK, DIM (0.8 mg·L^−1^), PA (0.1 mg·mL^−1^), and DIM (0.8 mg·L^−1^) + PA (0.1 mg·mL^−1^), respectively. (**A**–**F**) indicated that the relative expression level of *Ssodc1, Ssodc2, CWDE2, CWDE10*, *SsBil1* and *SsGgt1*.

The relative gene-expression levels of six pathogenic genes of *S. sclerotiorum* were assessed by qRT-PCR. It showed that DIM or PA treatment significantly improved relative expression levels of *Ssodc2, CWDE2, CWDE10*, while the *SsBil1* and *SsGgt1* gene expressions were down-regulated. Furthermore, compared with the control, the expression levels of *Ssodc2, CWDE2,* and *CWDE10* with DIM treatment were up-regulated by 950%, 660% and 250%, respectively (Figure 7B–D). Obviously, gene-expression levels of *SsGgt1* were decreased by 36.1% in DIM treatment (Figure 7F). Similarly, compared with control, PA and DIM + PA did evoke obvious increases in *Ssodc1, Ssodc2, CWDE2,* and *CWDE10* gene-expression levels, and PA up-regulated the expressions of these genes by 341% and 560%, 80.3% and 76%, respectively. DIM + PA up-regulated the expressions of these genes by 209% and 250%, 48.6% and 147%, respectively (Figure 7A–D). Differently, the relative gene-expression levels of *SsGgt1* with DIM and PA were decreased by 36.1% and 26.7%, while the expression level of *SsGgt1* with DIM + PA was increased by 33.4% (Figure 7F).

## 4. Discussion

### 4.1. PA Related-Metabolic Pathway of Rape Regulated by Se Was Relevant to Disease Resistance 

In recent years, fungicide application has still been the main method for controlling *S. sclerotiorum* [26,27]. Many fungicides with specific modes of action, including benzimidazole fungicides carbendazim, dicarboximide fungicides (DCFS) dimethachlon, and other groups, lack durability due to the development of resistance, as well as induced environmental pollution and food safety [28,29,30]. There is an urgent need to find new alternative fungicides with different modes of action for consistent control of *S. sclerotiorum*. Our previous research has proved that Se can reduce *S. sclerotiorum* incidence of rape by increasing plant Se concentration, shifting soil microbial community and functional profiles [31]. Another research study showed that in a field experiment, when sclerotia was retreated with Se (VI), the pathogenicity to oilseed rape leaves was reduced [32]. Interestingly, dissolved organic matter derived from rape straw pretreated with selenium in soil improves the inhibition of *S. sclerotiorum* growth, and metabolites up-regulated in DOM showed significant inhibition on *S. sclerotiorum* growth [20]. Therefore, plant-derived metabolites can be used as an environmentally friendly fungicide. In this study, Se regulated multiple metabolism pathways of rape straw (Figure 1A), and the phenylacetic acid in phenylalanine metabolism was one of the up-regulated metabolites as it could affect the content of phenylpropanoids in phenylalanine metabolism (Figure 1B). Phenylpropanoids are ubiquitous plant phenolics that occur in defense root exudates, which are often detected as biomarkers of fungal infections; research has found that resistance to Fusarium graminearum attack in barley is based on the rapid accumulation and secretion of phenylpropanoids (cinnamic acid derivatives) after fungal infection [33,34,35]. Additionally, the contents of fumarate, an intermediate in the TCA cycle, and succinate, an intermediate in the metabolism of alanine, aspartate and glutamate metabolism, were significantly affected by phenylacetic acid. A study on Brassica rape has shown high levels of fumarate and phenylpropanoids in fungus-infected plants, which is related to the effects of systemic resistance [20,36]. Therefore, the regulation of the PA and PA-related metabolic pathway by Se was beneficial in reducing the incidence rate of *S. sclerotiorum* (Figure 1B).

### 4.2. PA Reduced Application Amount of DIM and Enhanced Inhibition Effect for S. sclerotiorum 

PA is an intermediate in organic synthesis of medicine and pesticides [37], and itself is also a pesticide [38] and plant growth hormone [39,40]. It should be noted that PA is harmful when inhaled, ingested or absorbed through the skin. Therefore, the appropriate dosage should be considered in the actual process of use, and the toxicity test should be carried out in advance to avoid its toxicological harm. To compare the inhibitory effect of DIM and PA alone as well as using DIM and PA together on *S. sclerotiorum,* the baseline sensitivity of *S. sclerotiorum* to DIM and PA was established at first. The EC_50_ value of the distribution of populations was a key reference for evaluating resistance risk and monitoring resistance levels in the pathogen populations. In this research, *S. sclerotiorum* was tested for sensitivity to different concentrations of DIM and PA through testing the inhibition of mycelial growth. The EC_50_ value of DIM was greater than that of PA, which indicated that DIM has stronger resistance than PA (Figure 2A, B). Numerous researchers have reported that a variety of plant pathogens are resistant to DCFs [30]. 

The mycelial growth, weight of sclerotia in medium and the disease lesion diameter of rape leaves in vitro were measured using 0.8 mg·L^−1^ DIM and 0.1 mg·mL^−1^ PA, and the results showed that DIM or PA inhibited mycelial growth (Figure 3B). Compared with the treatment of 1 mg·L^−1^ DIM, the growth-inhibition rate was increased with 0.8 mg·L^−1^ DIM +0.1 mg·mL^−1^ PA, which indicated that PA can enhance the prevention of *S. sclerotiorum* by dimethachlon. Similar inhibition effects of fungicides such as flusilazole and demethylation inhibitors (DMIs) observed on hyphae growth and sclerotia development have been reported [41]. Phenylacetic acid is an organic chemical material. Studies have shown that the inhibition rate of filament fungi, *D. bryoniae,* was above 25% when treated with 0.1 mg·mL^−1^ phenylacetic acid [42]. Interestingly, the application of 0.8 mg·L^−1^ DIM and 0.1 mg·mL^−1^ PA obviously improved the *S. sclerotiorum*-inhibition rate and weight of sclerotia after 10 days compared to DIM and PA alone, which indicated that the use of DIM and PA was an effective method for inhibiting *S. sclerotiorum* and that PA could reduce the application amount of DIM. The formation of sclerotia requires appropriate external conditions, such as nutrition, light, pH, humidity and temperature, among which pH is the most critical factor affecting sclerotia formation and the pathogenicity of sclerotia [43]. In this study, compared with the control, the weight of sclerotia increased after 10 days of treatment. This may be due to the number of sclerotia increased in DIM and PA treatment (Figure 3C,D). 

### 4.3. The Inhibitory Effect Mechanism of PA and DIM on S. sclerotiorum

**Figure 8:**
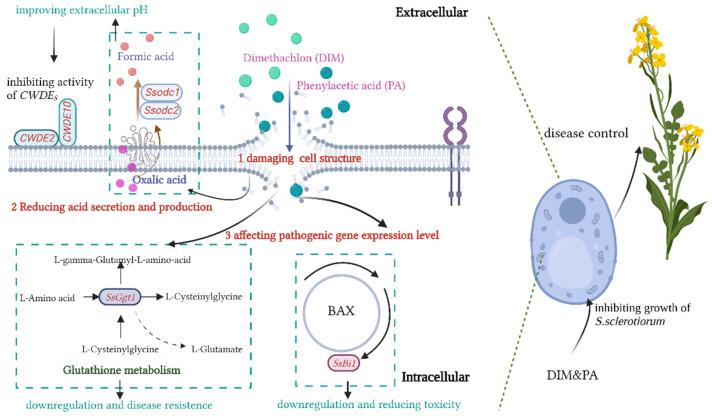
**Possible mechanisms regarding the role of DIM and PA in reducing the pathogenicity of *S. sclerotiorum***. (1) PA and DIM damaged the cell structure; (2) PA and DIM changed pathogenic factors and growth environment; (3) PA and DIM regulated the expression level of the pathogenicity gene.

When investigating the inhibition effects of DIM and PA on *S. sclerotiorum*, it might involve the following mechanistic processes (Figure 8):

(1) PA and DIM damaged the cell structure and inhibited the growth of *S. sclerotiorum*.

Previous studies have shown that fungicides (flusilazole and fludilamide) can change the surface morphology and internal structure of the mycelia [28,44]. In this study, morphological changes of mycelial cells were observed. Mycelia were found to be twisted and fractured after DIM treatment and twisted after PA treatment using scanning electron microscopy, and mycelia shrank and tore when DIM and PA were used together. Transmission electron microscopy showed that the cytoplasm contracted and aggregated and the cell wall deformed after DIM and PA treatment. When DIM and PA were used together, the cytoplasm was degraded and the cell wall was cracked. The inhibition of the fungicide on nuclear discs showed that fungicide induced more branches at the tip of the mycelia, which slows the spread of the fungus [41]. The main function of the cell membrane is to increase cellular permeability and participate in intracellular energy as a protein matrix, targeting signal transduction, solute transport and DNA activities such as replication [45]. Results indicated that DIM and PA play a role by destroying cell walls and reaching the cell membrane, thus promoting a series of changes in cells. Ion homeostasis not only plays an essential role in maintaining intracellular energy stability, but also plays an important role in solute transport, metabolic regulation, cell turgor and dynamic equilibrium [46]. When the integrity of the cell membrane is destroyed, leakage occurs in the cells [47].

(2) PA and DIM reduced pathogenicity of *S. sclerotiorum* by changing pathogenic factors and growth environment.

After DIM treatment, the conductivity increased with the time of mycelium immersion in deionized water, and the conductivity was significantly lower than that of the control after DIM treatment at any time (*p* < 0.05), which might be due to the resistance of sclerotinia to DIM. However, compared with control, only using PA or using DIM and PA together increased the conductivity, indicating that using PA alone and the use of DIM and PA together may promote electrolyte exosmosis in mycelium cells; this result is consistent with that of Zhou et al. (2014). Similarly, it was found that in the study of the inhibiting mechanism of sclerotinia, a chemical pesticide was used to prevent *S. sclerotiorum* and the leakage of electrolytes in the cell membrane of *S. sclerotiorum* [29]. pH is the most critical factor affecting sclerotia formation and the pathogenicity of sclerotinia [43]. In this study, acid production was decreased after treatment, which induced the improvement of pH and inhibited the pathogenicity of sclerotinia. Oxalic acid is a major pathogenic factor for sclerotinia [48]. OA secretion was reduced obviously after treatment in our study, which also explained that acid production had decreased. The study showed that the pH of the surrounding environment around the infection site is required to determine whether pathogens can successfully infect host plants [24,49]. Importantly, we found that disease lesion diameter was remarkably correlated linearly with OA secretion (R^2^ = 0.951) (Figure 6), which also illustrated that OA secretion is a direct pathogenic factor.

(3) PA and DIM regulated the expression level of the pathogenicity gene and affected the pathogenicity of *S. sclerotiorum.*

Recently, the roles of OA and cell wall-degrading enzymes (CWDEs) have been focused on the molecular aspects of the pathogenicity of *S. sclerotiorum* [50]. CWDEs can help with the penetration and degradation of host cell walls [51]. *Odc1* and *Odc2* are two putative oxalate decarboxylase genes involved in oxalic acid degradation [52]. Compared with the control, the relative expression levels of *Ssodc1* and *Ssodc2* were increased in our study, which further explained the decrease in OA secretion. The cell wall takes the lead in limiting the infiltration of pathogens and the spread barrier of infection [53]. CWDEs are an essential extracellular enzyme secreted by pathogens, and they can degrade plant cell walls and penetrate plant tissue for infection [54], however, *CWDE2* and *CWDE10* were increased after treatment. Up-regulated CWDE gene expression was associated with low virulence [55]. Similarly, Xu et al. (2015) found that the increase of the *CWDE* gene did not promote the spread of *sclerotinia* on pea leaves [24]. This may be due to CWDE being influenced by the environment, such as high-pH conditions [56]. In spite of OA and CWDES, *Ggt1* and *Bi1* were related to pathogenic factors. Yu et al. (2015) has proved that the *Bi1* code was a hypothetical BAX inhibitory protein. The protein can induce the full toxicity of sclerotinia [57]. The expression level of *SsBil1* was decreased obviously after using DIM alone as well as using DIM and PA together, which reduced the pathogenicity of *S. sclerotiorum.* The expression level of *SsBil1* showed no significant difference, which might be because PA changed the pH of the environment. In addition, *SsGgt1* (Gamma glutamyl transpeptidase) is the regulation of the antioxidation system of sclerotinia [58]. The expression level of *SsGgt1* was decreased after DIM and PA treatment, while there was no significant difference after using DIM and PA together. Recent research has reported that some secretory proteins related to their virulence do exist in sclerotinia, which may affect the gene-expression levels of *SsGgt1* [59]. 

## 5. Conclusions

The Se-mediated PA-related metabolic pathway improved the PA content in oilseed rape and reduced the incidence rate of *S. sclerotiorum*. PA significantly inhibited mycelial growth and pathogenicity as well as enhanced the growth inhibition of *S. sclerotiorum* by DIM. The mycelia morphological structure damage response of DIM was increased by PA. DIM changed the pathogenic factors of *S. sclerotiorum*, and PA enhanced the changes induced by DIM. Further studies revealed that PA and DIM applications regulated the expression level of pathogenic genes. The findings will be helpful to provide evidence that PA has promising potential as an alternative to novel fungicides for sclerotinia in oilseed rape.

## Figures and Tables

**Table 1:** Primers used for quantitative real-time PCR of target genes [20].

Gene Name	Forward Primer (5′-…-3′)	Reverse Primer (5′-…-3′)
*Ssodc1*	GATGAGGGCCAACTTACGGT	CGGGAGTGTTAGGCTTCAGG
*Ssodc2*	ATTTCTTGCCAACGCCCAAC	TGGGCCACGTAAGTTTGGAA
*CWDE2*	ATGCTCCTTTCACCACAACC	ACACCCCCATTCGCATAATA
*CWDE10*	CCAATGGAGATCAGCAAGGT	TTGTACATTCGCCAAACCAA
*SsBi1*	TCATTCCAGCAAACCTACAACC	GAACATAGCACCACCCACGAG
*SsGgt1*	AGATCGCCCAACTTCACTCG	TTCCCTCTGTCAAAGTCGCC
*β-tublin*	TTGGATTTGCTCCTTTGACCAG	AGCGGCCATCATGTTCTTAGG

**Table 2:** KEGG pathway of different Metabolite.

Pathway	Description	# Compounds (dem)	Compounds (dem)	# Compounds (All)	Compounds (All)
brp01100	Metabolic pathways-Brassica rapa (field mustard)	36	C00049; C00407; C00042; C00257; C00122; C00106; C00334; C00818; C00299; C00294; C00212; C00217; C00333; C00047; C07086; C00253; C00198; C06337; C00242; C00188; C00547; C00689; C00954; C05422; C00184; C00568; C06104; C02226; C00077; C01762; C00438; C06087; C06424; C00449; C06552; C00064	124	C00183; C00180; C00037; C00049; C00148; C00189; C00407; C00042; C00137; C00257; C00989; C00122; C05519; C00249; C01236; C00794; C00106; C00149; C00334; C01904; C00818; C00258; C00299; C00294; C00178; C00212; C00158; C00217; C01083; C00333; C00047; C01234; C01595; C00208; C07086; C00243; C00387; C01571; C01494; C00093; C00116; C00253; C00055; C00327; C02273; C00209; C06423; C06156; C06427; C00198; C06337; C00124; C00059; C00099; C00233; C00191; C00199; C00232; C00089; C00811; C00160; C00847; C00493; C00242; C00559; C00188; C01013; C00547; C00147; C01732; C00263; C00295; C00689; C00026; C00379; C00954; C00499; C01514; C01089; C00979; C01040; C00836; C04411; C05422; C00319; C00090; C00184; C02656; C00624; C00036; C01157; C00735; C00601; C00590; C00568; C00530; C01717; C06104; C00152; C02226; C00077; C01762; C10164; C00438; C06087; C00509; C03722; C01617; C05953; C06424; C05635; C00449; C01042; C05437; C00016; C00581; C02512; C06555; C05141; C00385; C06712; C06552; C00051; C00064
brp01110	Biosynthesis of secondary metabolites-Brassica rapa (field mustard)	13	C00049; C00407; C00042; C00257; C00122; C00047; C00253; C00198; C00188; C00077; C01762; C06087; C00449	50	C00183; C00180; C00037; C00049; C00148; C00407; C00042; C00257; C00122; C01236; C00149; C00258; C00158; C01083; C00047; C01234; C01494; C00093; C00253; C00327; C06427; C00198; C00099; C00233; C00199; C00811; C00160; C00493; C00188; C00263; C00026; C01514; C00979; C04411; C00196; C00624; C00036; C01479; C00590; C00152; C00077; C01762; C06087; C00509; C05901; C01617; C00449; C00016; C02512; C00385
brp02010	ABC transporters-Brassica rapa (field mustard)	9	C00049; C00407; C00121; C00333; C05402; C00047; C00188; C00077; C00064	28	C00183; C00037; C00049; C00148; C00407; C00137; C00121; C00794; C00487; C01083; C00333; C05402; C00047; C00208; C00243; C00093; C00392; C00116; C02273; C00059; C00089; C01835; C00188; C00379; C00492; C00077; C00051; C00064
brp01230	Biosynthesis of amino acids-Brassica rapa (field mustard)	7	C00049; C00407; C00047; C00188; C00077; C00449; C00064	22	C00183; C00037; C00049; C00148; C00407; C00158; C00047; C00327; C00233; C00199; C00493; C00188; C00263; C00026; C00979; C04411; C00624; C00036; C00152; C00077; C00449; C00064
brp00250	Alanine, aspartate and glutamate metabolism-Brassica rapa (field mustard)	6	C00049; C00042; C00122; C00334; C00438; C00064	12	C00049; C00042; C00122; C00334; C00158; C00232; C00026; C00036; C00152; C00438; C01042; C00064
brp00760	Nicotinate and nicotinamide metabolism-Brassica rapa (field mustard)	6	C00049; C00042; C00122; C00334; C01384; C00253	8	C00049; C00042; C00122; C00334; C01384; C00253; C00232; C03722
brp01200	Carbon metabolism-Brassica rapa (field mustard)	6	C00049; C00042; C00257; C00122; C00198; C00184	20	C00037; C00049; C00042; C00257; C00989; C00122; C01236; C00149; C00258; C00158; C00198; C00199; C00232; C00160; C01013; C01732; C00026; C00979; C00184; C00036
brp00230	Purine metabolism-Brassica rapa (field mustard)	5	C00294; C00212; C00242; C01762; C00064	12	C00037; C00294; C00212; C00387; C00059; C00242; C00559; C00147; C00499; C01762; C00385; C00064
brp00970	Aminoacyl-tRNA biosynthesis-Brassica rapa (field mustard)	5	C00049; C00407; C00047; C00188; C00064	9	C00183; C00037; C00049; C00148; C00407; C00047; C00188; C00152; C00064
brp01210	2-Oxocarboxylic acid metabolism-Brassica rapa (field mustard)	5	C00049; C00407; C00047; C02226; C00077	12	C00183; C00049; C00407; C00158; C00047; C00233; C00026; C04411; C00624; C00036; C02226; C00077
brp00053	Ascorbate and aldarate metabolism-Brassica rapa (field mustard)	4	C00879; C00818; C00333; C05422	8	C00137; C00879; C00818; C00333; C00191; C00026; C01040; C05422
brp00220	Arginine biosynthesis-Brassica rapa (field mustard)	4	C00049; C00122; C00077; C00064	7	C00049; C00122; C00327; C00026; C00624; C00077; C00064
brp00240	Pyrimidine metabolism-Brassica rapa (field mustard)	4	C00106; C00299; C00438; C00064	10	C00106; C00299; C00178; C00055; C00099; C01013; C00295; C00526; C00438; C00064
brp00350	Tyrosine metabolism-Brassica rapa (field mustard)	4	C00042; C00122; C01384; C00547	8	C00042; C00122; C01384; C00232; C00811; C00547; C00642; C00530
brp00380	Tryptophan metabolism-Brassica rapa (field mustard)	4	C00954; C02693; C02172; C05831	10	C00954; C02043; C02470; C02693; C01717; C10164; C02172; C03722; C05635; C05831
brp00410	beta-Alanine metabolism-Brassica rapa (field mustard)	4	C00049; C00106; C00334; C05670	8	C00049; C00106; C00334; C00099; C01013; C01073; C05670; C03722
brp00650	Butanoate metabolism-Brassica rapa (field mustard)	4	C00042; C00122; C00334; C01384	8	C00042; C00989; C00122; C00334; C01384; C00232; C00026; C01089
brp00030	Pentose phosphate pathway-Brassica rapa (field mustard)	3	C00257; C00121; C00198	7	C00257; C00121; C01236; C00258; C00198; C00199; C03752
brp00290	Valine, leucine and isoleucine biosynthesis-Brassica rapa (field mustard)	3	C00407; C00188; C02226	6	C00183; C00407; C00233; C00188; C04411; C02226
brp00300	Lysine biosynthesis-Brassica rapa (field mustard)	3	C00049; C00047; C00449	5	C00049; C00047; C00263; C00026; C00449
brp00330	Arginine and proline metabolism-Brassica rapa (field mustard)	3	C00334; C01877; C00077	9	C00148; C00334; C01877; C05942; C01157; C00077; C00431; C00581; C05147
brp00360	Phenylalanine metabolism-Brassica rapa (field mustard)	3	C00042; C00122; C07086	8	C00180; C00042; C00122; C07086; C00811; C00642; C00601; C02137
brp00460	Cyanoamino acid metabolism-Brassica rapa (field mustard)	3	C00049; C00407; C05670	8	C00183; C00037; C00049; C00407; C05670; C00152; C01401; C02512
brp00960	Tropane, piperidine and pyridine alkaloid biosynthesis-Brassica rapa (field mustard)	3	C00407; C00047; C00253	4	C00407; C00047; C00253; C01479
brp00020	Citrate cycle (TCA cycle)-Brassica rapa (field mustard)	2	C00042; C00122	6	C00042; C00122; C00149; C00158; C00026; C00036
brp00190	Oxidative phosphorylation-Brassica rapa (field mustard)	2	C00042; C00122	2	C00042; C00122
brp00260	Glycine, serine and threonine metabolism-Brassica rapa (field mustard)	2	C00049; C00188	7	C00037; C00049; C05519; C00258; C00188; C00263; C00581
brp00261	Monobactam biosynthesis-Brassica rapa (field mustard)	2	C00049; C00188	3	C00049; C00059; C00188
brp00310	Lysine degradation-Brassica rapa (field mustard)	2	C00047; C00449	7	C00037; C00487; C00047; C00489; C00449; C02727; C00431
brp00480	Glutathione metabolism-Brassica rapa (field mustard)	2	C05422; C00077	4	C00037; C05422; C00077; C00051
brp00620	Pyruvate metabolism-Brassica rapa (field mustard)	2	C00042; C00122	4	C00042; C00122; C00149; C00036
brp00630	Glyoxylate and dicarboxylate metabolism-Brassica rapa (field mustard)	2	C00042; C00064	12	C00037; C00042; C00149; C00258; C00158; C00209; C00160; C01732; C00026; C00036; C00898; C00064
brp00660	C5-Branched dibasic acid metabolism-Brassica rapa (field mustard)	2	C02341; C02226	5	C00490; C02341; C01732; C00026; C02226
brp00770	Pantothenate and CoA biosynthesis-Brassica rapa (field mustard)	2	C00049; C00106	4	C00183; C00049; C00106; C00099
brp00910	Nitrogen metabolism-Brassica rapa (field mustard)	2	C00192; C00064	2	C00192; C00064

**Table 3:** Combined inhibition of different concentration of DIM and PA on *S. sclerotiorum* growth.

Treatments			Mycelium Growth (cm)	Inhibitory Ratio (%)	Notes
Name	DIM Concentration (mg/L)	PA Concentration (mg/mL)			
CK1	None	None	6.09 ± 0.00a	/	Previous study [21]
T1	1.0 mg/L DIM	None	3.96 ± 0.14b	35.50%	
T2		0.10 mg/mL PA	1.92 ± 0.09c	68.73%	
CK2	None	None	5.28 ± 0.03a	/	This study
T3	0.8 mg/L DIM	None	3.91 ± 0.07d	25.82%	
T4		0.10 mg/mL PA	2.50 ± 0.00e	52.61%	
Name	Reduced DIM content (mg) (1L solution)	Increased PA content (g) (1L solution)	Δ Mycelium growth (cm)	Increased Inhibitory ratio (%)	Calculated by data of previous study and this study
T4/T1	0.20	0.10	2.13/2.78	17.11%	

Notes: Data were analyzed by one-way ANOVA and shown as means ± SD. Different letters indicate statistically significant differences within the same concentration of DIM (*p* < 0.05). T4/T1 means that compared with T1, there is a variation in DIM content, PA content, mycelium growth and inhibitory ratio in T4.

## Data Availability

Not applicable.
